# The Microbial Community of the Respiratory Tract of Commercial Chickens and Turkeys

**DOI:** 10.3390/microorganisms10050987

**Published:** 2022-05-08

**Authors:** Olimpia Kursa, Grzegorz Tomczyk, Karolina Adamska, Justyna Chrzanowska, Anna Sawicka-Durkalec

**Affiliations:** 1 Department of Poultry Diseases, National Veterinary Research Institute, Al. Partyzantów 57, 24-100 Puławy, Poland; gtomczyk@piwet.pulawy.pl (G.T.); karolina.adamska@piwet.pulawy.pl (K.A.); anna.sawicka@piwet.pulawy.pl (A.S.-D.); 2 Veterinary Practice Kokovet, Kanta 42/9, 10-691 Olsztyn, Poland; justyna.chrzanowska@animal-pharma.com

**Keywords:** respiratory tract, turkey, chicken, bacterial composition

## Abstract

Respiratory tract health critically affects the performance of commercial poultry. This report presents data on the microbial community in these organs from a comprehensive study of laying chickens and turkey breeders. The main objective was to characterize and compare the compositions of the respiratory system bacteria isolated from birds of different ages and geographical locations in Poland. Using samples from 28 turkey and 26 chicken flocks, the microbial community was determined by 16S ribosomal RNA sequencing. There was great variability between flocks. The diversity and abundance of upper respiratory tract (URT) bacteria was greater in chickens than in turkeys. At the phyla level, the URT of the chickens was heavily colonized by Proteobacteria, which represented 66.4% of the total microbiota, while in turkeys, this phylum constituted 42.6% of all bacteria. Firmicutes bacteria were more abundant in turkeys (43.2%) than in chickens (24.1%). The comparison of the respiratory tracts at the family and genus levels showed the diversity and abundance of amplicon sequence variants (ASV) differing markedly between the species. Potentially pathogenic bacteria ASV were identified in the respiratory tract, which are not always associated with clinical signs, but may affect bird productivity and performance. The data obtained, including characterization of the bacterial composition found in the respiratory system, may be useful for developing effective interventions strategies to improve production performance and prevent and control disease in commercial laying chickens and turkeys.

## 1. Introduction

The avian respiratory tract is a primary route for infections by many pathogens causing chronic diseases, and among all such diseases, those with respiratory involvement are the main cause of economic losses in the poultry industry [1,2,3]. The high popularity of poultry meat among consumers, its nutritional value along with attractive price prompt the conduct of extensive studies on how a favorable poultry microbiome is constituted. Research with metagenomic sequencing has focused primarily on the microbes in the gastrointestinal tract and their metabolic and immune functions [4,5,6]. In a number of recent differently designed research studies using various study populations, sequencing was also used to survey the chicken respiratory microbiota [7,8,9]. The overall results support the notion that the respiratory microbiota is of paramount importance to poultry respiratory health and imply that better knowledge of microbial succession in the respiratory tract and its dysregulation in infection could provide crucial understanding of the pathophysiology of respiratory infections.

Economic losses in the poultry industry can be caused by horizontally transmitted pathogens. Direct conduct between contaminated materials and susceptible animals can spread infection, which invades the respiratory tract and spreads among birds kept in the same poultry house [1,10,11]. Infections of the poultry respiratory tract involve complex interactions between the host, pathogen, environment, and management factors [12,13,14]. In addition, viral infections can increase susceptibility to bacterial infections either by immunosuppression or by damaging the epithelium of upper airways such as to favor the colonization of the lower respiratory tract by bacterial pathogens [15,16]. Similar environmental factors or copathogen presence in respiratory tract disorder the microbiota of birds, causing dysfunction, and may allow the growth of pathogenic bacteria already present [12,17,18].

Many pathogens of the respiratory tract are major risk factors in poultry health. In many cases, the clinical form of the disease which a pathogen causes is not observed universally, and the infected birds without manifestation of disease remain carriers of the pathogen. Many infections most frequently occur as chronic and subclinical infections, such as mycoplasmosis or ornithobacteriosis affecting the upper respiratory tract (URT) [19,20,21]. Respiratory diseases can, however, also cause serious clinical signs [22,23,24], which in many cases may be intensified by the presence of other environmental factors or pathogens [25,26]. Where disease has a copathogenic aspect, higher morbidity and mortality are observed in mixed bacterial infections [11,27,28]. Respiratory distress, airsacculitis, sinusitis, nasal discharge, sneezing, and facial edema are typical indicia of avian bacterial respiratory disease and other signs may also include depression, lower food intake, reduced weight gains and egg production, decreased growth and higher mortality.

In the present study, we characterized and compared the composition of the bacterial community of the respiratory tract of commercial chickens and turkeys. The birds were at different ages and came from different regions of Poland. Assessment of the bacterial diversity in the respiratory tracts of poultry expands our understanding of the chicken and turkey microbiota as well as of the colonization of the trachea by microorganisms. We hope that the data gathered will contribute a resource for the planning of the most effective way to monitor poultry flocks.

## 2. Materials and Methods

### 2.1. Sample Collection

Samples were collected from birds from commercial farms delivered to the National Veterinary Research Institute in Poland (NVRI) as part of diagnostic tests at different time points. The birds were raised on a floor with wood shavings as litter and provided ad libitum access to feed and water. Shortly after delivery to the NVRI, tracheal samples were taken from the birds with a sterile swab under aseptic conditions and the swabs were placed in sterile tubes. Samples were collected from 30 chicken flocks (5 birds per flock; *n* = 150) and 30 turkey flocks (5 birds per flock; *n* = 150). The birds were of different ages and came from different regions of the country (Table 1). A total of 300 swab samples were collected and used for 16S ribosomal RNA (rRNA) gene analysis. Summary information on the flocks, ages of birds, and geographical locations of the farms is shown in Table 1. All swab samples were mixed with Tris buffer (10 mM, pH 8.5; Eurx, Gdańsk, Poland) and kept at −20 °C until DNA extraction.

### 2.2. DNA Extraction

DNA was extracted using a Maxwell RSC PureFood Pathogen Kit (Promega, Madison, WI, USA) according to the manufacturer’s recommendations. The negative control in the DNA extraction was the Tris buffer used for sample preparation. In the isolation step, 50 µL of lysozyme (10 mg/mL, Novazym, Poznań, Poland), 6 µL of mutanolysin (5 kU/mL, Sigma-Aldrich, St. Louis, MO, USA), and 8 µL of lysostaphin (5 g/mL, Sigma-Aldrich) were added to the samples and the mixtures were incubated for 45 min at 37 °C. The quantity and quality of the DNA was determined using the Nanodrop 1000 system (Thermo Fisher Scientific, Waltham, MA, USA).

### 2.3. 16S rDNA Sequencing

After extracting DNA, the V3–V4 hypervariable regions of the 16S rRNA gene were amplified and a library was prepared. Amplicon libraries were created using 341f/785r primers generating amplicons of ~440 bp [29]. A PCR reaction was performed using Q5 Hot Start High-Fidelity 2× Master Mix (New England BioLabs, Ipswich, MA, USA) with reaction conditions as recommended by the manufacturer. Quantification of the libraries was carried out with use of a Qubit 3.0 fluorometer (Thermo Fisher Scientific). Normalization of the libraries was performed according to the Illumina protocol. Sequencing of samples was performed using MiSeq paired-end 2 × 300 bp technology in a v3 kit as per the manufacturer’s direction (Illumina, San Diego, CA, USA).

### 2.4. Analysis of Microbial Composition

Raw reads (obtained after sequencing) were subjected to quality control in Cutadapt software, where adapter, primer sequences and low quality bases were removed [30]. During this step, the chimeric sequences were filtered and trimmed. Sequences were processed and taxonomy assigned using QIIME2 [31]. ASV were determined with DADA2 using the denoise-paired method. The taxonomic assignment was done with the use of SILVA 138 [32,33]. We filtered out all ASV that belonged to Chloroflexi and Cyanobacteria and all ASV that occurred in fewer than three samples.

### 2.5. Statistical Analysis

Statistical analysis was carried out using the phyloseq and vegan packages of R [34,35,36]. Alpha diversity was measured using the Shannon and Chao1 indices, and per-age- group statistical comparisons of observed richness were conducted. A beta diversity heatmap was generated based on the Bray–Curtis method [37]. Graphs were created using the heatmaply packages of R [38,39,40,41]. Venn diagrams were constructed showing the number of taxa at the phylum, family, and genus levels [42], and one way ANOVA was used to detect significant differences between assigned taxa. The Kruskal–Wallis test was performed on samples from chickens and turkeys for statistically significant differences in alpha diversity values. The value of *p* < 0.05 was considered statistically significant.

## 3. Results

### 3.1. DNA Extraction and Sequencing

We sequenced the bacterial communities of the URT of the swabbed chickens and turkeys. Good quality DNA allowing further analysis was obtained from 28 chicken flocks and 26 turkey flocks out of 30 sampled flocks in each case.

### 3.2. Microbial Composition of the Chicken URT

**Figure 1:**
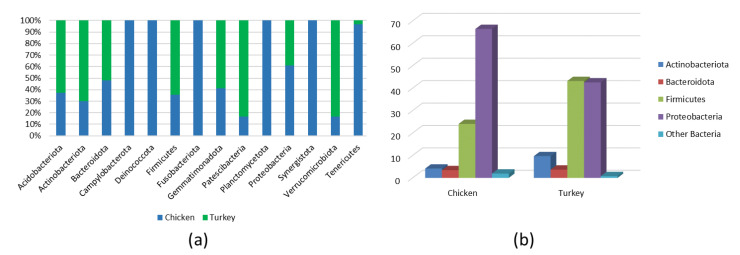
(**a**) All phyla in chicken and turkey URTs. (**b**) Main phyla in chicken and turkey URTs.

**Figure 2:**
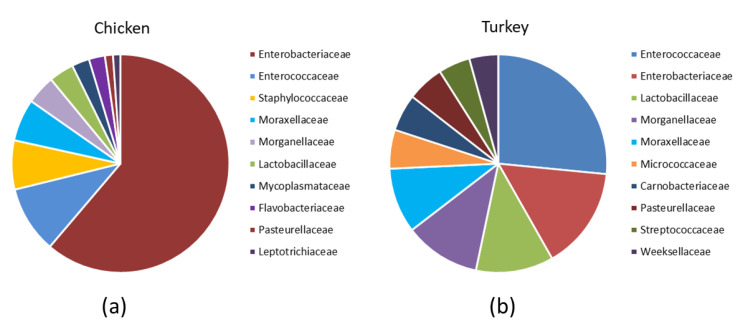
Relative abundance of the 10 most prevalent families among the URT bacteria in (**a**) chickens and (**b**) turkeys.

**Figure 3:**
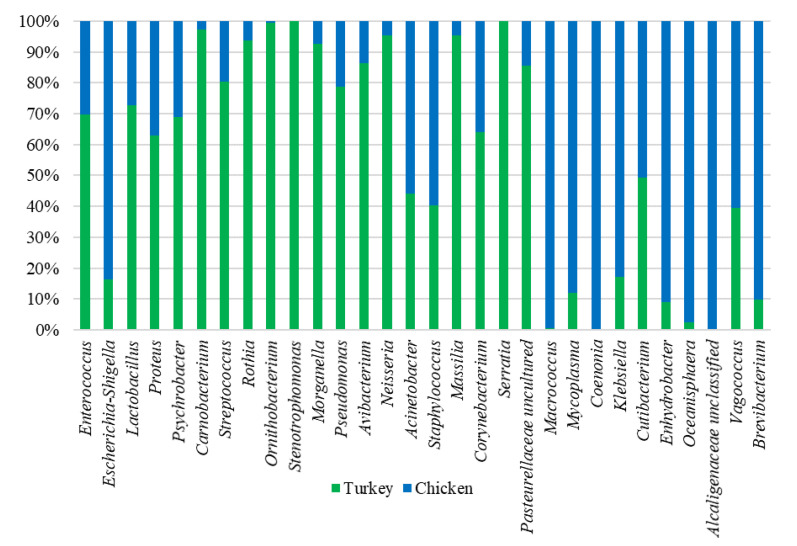
Relative abundance of the 20 most present genus in chicken and turkey URTs. Some genera occur in only one of the species.

Analysis of the bacterial diversity of the chicken URT showed that the dominant phyla were Proteobacteria, containing 66.4% of isolates, and Firmicutes containing 24.06%, Actinobacteriota with 4.16% and Bacteroidota with 3.45% also being well represented (Figure 1a,b). At the family level, the chicken URT bacterial community was dominated by *Enterobacteriaceae* (52.8%). The other taxa at the family level comprising the most prevalent 10 were, with their percentages of bacteria, *Enterococcaceae*, 8.57%; *Staphylococcaceae*, 6.27%; *Moraxellaceae*, 5.39%; *Morganellaceae*, 3.72%; *Lactobacillaceae*, 3.2%; *Mycoplasmataceae*, 2.25%; *Flavobacteriaceae*, 2.05%; *Pasteurellaceae*, 1.03% and *Leptotrichiaceae*, 0.95% (Figure 2a and Supplementary Table S1). At the genus level, *Escherichia–Shigella* and *Enterococcus* were the most heavily present. The *Proteus, Macrococcus, Lactobacillus, Staphylococcus, Psychrobacter, Mycoplasma, Acinetobacter, Coenonia* and *Klebsiella* genus were lower in abundance (but higher than 1%). The *Coenonia* and unclassified *Alcaligenaceae* were present only in chickens (Figure 3 and Supplementary Table S2).

### 3.3. Microbial Composition of the Turkey URT

An analysis of phyla showed that turkey URT isolates were dominated by Firmicutes, 43.2% of the bacteria being of this phyla, and Proteobacteria, to which 42.6% of the pathogens were affiliated (Figure 1a,b). The 10 most abundant families and the corresponding percentages of bacteria were *Enterococcaceae*, 19.66%; *Enterobacteriaceae*, 11.28%; *Lactobacillaceae*, 8.55%; *Morganellaceae*, 8.36%; *Moraxellaceae*, 7.16%; *Micrococcaceae*, 4.27%; *Carnobacteriaceae*, 4.07%; *Pasteurellaceae*, 4.05%; *Streptococcaceae*, 3.5% and *Weeksellaceae*, 3.18% (Figure 2b and Supplementary Table S3). The most dominant genus in the respiratory tracts of turkey were *Enterococcus*, *Escherichia–Shigella* and *Lactobacillus*. Next on the genus level with abundance higher than 1% were *Proteus*, *Psychrobacter*, *Carnobacterium*, *Streptococcus*, *Rothia*, *Ornithobacterium*, *Stenotrophomonas*, *Morganella*, *Pseudomonas*, *Avibacterium*, *Neisseria*, *Acinetobacter*, *Staphylococcus*, *Massilia*, *Corynebacterium*, *Serratia* and unclassified *Pasteurellaceae.* Of these, *Serratia* and *Stenotrophomonas* were not found in chickens (Figure 3 and Supplementary Table S4).

### 3.4. Comparison of the Compositions of the Microbial Communities of Chicken and Turkey URTs

**Figure 4:**
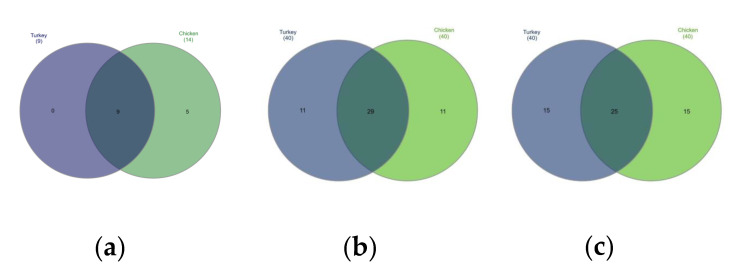
Venn diagrams showing shared taxa among the 40 most common at the: (**a**) phyla level, (**b**) family level, and (**c**) genus level.

The bacterial composition of the URT at the phylum level in both chickens and turkeys was dominated by Proteobacteria, Firmicutes, Bacteriodota and Acinobacteria. In turkeys, Proteobacteria and Firmicutes were at similar levels, while in chickens, Proteobacteria predominated (Figure 1b). Actinobacteriota were more abundant in the URTs of turkeys, while in chickens, bacteria of this phylum were similarly abundant to those of Bacteroidota (Figure 1b). Out of the 14 phyla present in chickens, 9 were also present in turkeys. Those that were found only in chickens were *Deinococcota*, *Campylobacterota*, *Fusobacteriota*, *Planctomycetota* and *Synergistota* (Figure 1a and Figure 4a). 

Differences in the URT microbial communities of chickens and turkeys were further analyzed at the family and genus level. Of the 40 most successfully colonizing families, 29 were common to chickens and turkeys. The other 11 families were at very different abundances in the two species or unique to one (Figure 4b). *Enterobacteriaceae* was the dominant family in chickens (Figure 2a and Supplementary Table S1), while in turkeys, this family was second to *Enterococcaceae* (Figure 2b and Supplementary Table S3). The chicken colonizing families included *Leptotrichiaceae*, *Flavobacteriaceae*, *Mycoplasmataceae* and *Staphylococcaceae* among the 10 most present; however, these families were not in the equivalent 10 which colonized turkeys. In turkeys, the *Micrococcaceae*, *Carnobacteriaceae*, *Streptococcaceae* and *Weeksellaceae* families were prominent, but they were not prominent in chickens (Figure 2b). 

When analyzing at the genus level, among the 40 genus in the highest abundance, 25 were common to both poultry species (Figure 4c). The 20 most common genus in turkeys and chickens are shown in Figure 3. Some were found in chickens in higher abundance than in turkeys, such as *Macrococcus*, *Mycoplasma*, *Klebsiella*, *Cutibacterium*, *Enhydrobacter*, *Oceanisphaera*, *Vagococcus* and *Brevibacterium.* Others such as *Coenonia* and unclassified *Alcaligenaceae* were present only in chickens. The most frequently detected bacterial genus in chickens were *Carnobacterium*, *Rothia*, *Ornithobacterium*, *Morganella*, *Avibacterium*, *Neisseria*, *Massilia* and uncultured *Pasteurellaceae* (Figure 3). *Serratia* and *Stenotrophomonas* were only found in turkeys.

**Figure 5:**
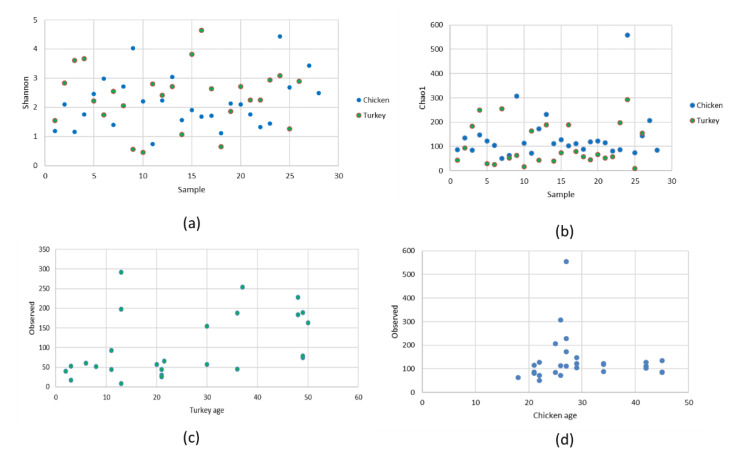
Alpha diversity comparison based on the: (**a**) Shannon index, (**b**) Chao1 index, (**c**) observed ASV in turkeys, and (**d**) observed ASV in chickens.

**Figure 6:**
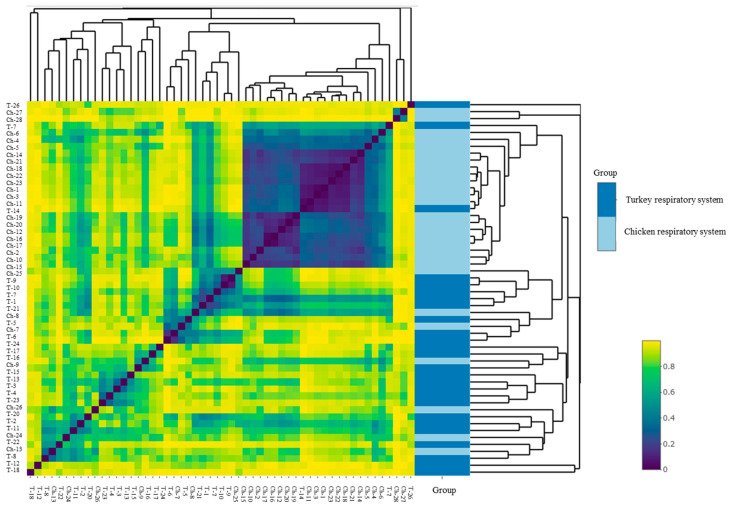
Beta diversity heatmap of chicken and turkey URT bacterial communities generated with the Bray–Curtis method.

The alpha diversity of the URT microbial communities was measured using Shannon and Chao1 indices. The Shannon index showed a very diverse range of species in both groups of birds (Figure 5a). No significant difference was found between the index in hens and the index in turkeys. Different numbers of microbial species in chicken URTs and turkey URTs was confirmed by the Chao1 index (*p* < 0.05, Kruskal–Wallis test) (Figure 5b). In turkeys, the number of observed ASV was progressively higher from 30 weeks of age (Figure 5c). In chickens, a parallel increase was noted but earlier, between 20 and 30 weeks of age (Figure 5d). The beta diversity of the microbial communities was measured by unweighted distance and Bray–Curtis metrics. A heatmap was compiled comparing species of community and it showed the differences between URT samples from chickens and turkeys (Figure 6).

## 4. Discussion

In this study, we focused on comparing the bacterial composition of the URT of chickens and turkeys from commercial poultry farms. The microbial colonization of the respiratory tract is a complex process, and can be shaped by several factors such as host genetics, age, antimicrobial use, vaccinations, season, different management strategies during the production cycle, and the surrounding environment. Additional factors may be the stress of moving the flock, the commencement of egg production or the composition of the diet [7,12,18,43,44,45]. The situation is similar for many other livestock such as cattle and pigs [46,47]. The current state of knowledge about poultry respiratory microbiota is limited only on the microbial composition of the chicken respiratory tract. Although the microbial community of the respiratory system in chickens has already been described, it has not been compared with that of turkeys in any investigation of a similar number of samples from both species. To the best of our knowledge, information on the respiratory microbiota of turkeys is currently inextensive and presented in only two studies [48,49]. This study extends knowledge of the bacteria inhabiting the respiratory system of turkeys and compares them to those in the respiratory system of chickens. The results of this investigation of the bacterial composition of chicken and turkey URTs correspond with those of our previous preliminary studies on these organs in turkeys and agree with studies conducted on chickens. Comparing results from multiple studies to ours, the microbial composition of the respiratory system in chickens is shown in all works to be similar at the phylum level.

The bacterial structure in the URT of turkeys differs from that of chickens. Despite the structural difference, some bacterial taxa at the genus and lower levels were common to turkeys and chickens. Our study shows that the bacterial composition of the respiratory tract of chickens is more diverse than that of turkeys (Figure 4a and Figure 5). The alpha diversity from abundance data was significantly different between chickens and turkeys (Figure 5b), although no additional statistically significant differences were observed. At the genus level, taking into account the 20 with the highest abundance of ASV in turkeys, there was only some overlap with those present in chickens. Several bacterial taxa were unique to chickens, just as there were taxa unique to turkeys (Figure 3 and Figure 4c). This may have been related not only to bird species, but also to age (Figure 5c,d). Results from studies conducted on other laying breeds differ in bacterial abundance and diversity from those obtained in this study. In Novogen Brown laying chickens studied in the UK, the predominant genus were *Staphylococcus*, *Enterobacteriaceae* and unclassified *Lactobacillus*, while in Hy-line W-36 breed laying chickens in the USA they were *Burkholderiaceae*, *Enterococcus*, *Lactobacillus*, *Rothia*, *Avibacterium*, *Gallibacterium* and *Mycoplasma* [7,12,18]. In our studied chickens, the dominant genera were also *Enterococcus*, *Lactobacillus*, *Staphylococcus* and *Mycoplasma*; however, *Escherichia–Shigella* was another dominant genus alongside them. The *Coenonia* genus was one of the 20 most present genera found only in this poultry species. Interestingly, *Coenonia* is a recently described genus that contains a novel single species—*C. anatine*—responsible for respiratory disease in ducks and geese [50].

The microbial composition of the URT in turkeys was made up in large part by *Enterococcus*, *Escherichia–Shigella* and *Lactobacillus*, and it differed slightly from the composition observed in other studies in turkeys where *Burkholderiaceae, Escherichia* and *Lactobacillus* were dominant. These genera were noted in chickens; however, genera that are only found in this poultry species such as *Serratia* and *Stenotrophomonas* were also observed. The *Serratia* genus contains specific respiratory commensal bacteria such as *S. marcescens*, which can promote infections with pathogens, of which *Mycoplasma gallisepticum* and *Ornithobacterium rhinotracheale* are two well-known examples [51]. The genus *Stenotrophomonas* contrasts by containing opportunistic bacteria that are widespread in the environment, including in food. Some of these bacteria are nosocomial pathogens that mainly cause respiratory infections in humans [52]. The differences between the observations of studies carried out in other laying breeds and other geographical areas highlight the need to gather comprehensive metadata for the analysis and comparison of research results.

Recent studies indicate that poultry production parameters are highly dependent on the correct composition of the gut microbiota [43,47]. Considering the hypothesis that some taxa present in the gastrointestinal tract may migrate to the respiratory tract in the form of aerosols in the respiratory system, it can be concluded that ASV of bacteria are present to a significant extent in the gut [49]. Changes in the abundance of taxa are largely related to the occurrence of various factors like stress associated with relocation to another poultry house or farm, temperature, and ventilation [45,53]. Environmental factors such as farm management and feeding may also instigate changes in microbiota constituent abundance [12,54]. The same factors affecting the abundance of certain taxa may be associated with the emergence of infections.

Knowledge of the respiratory microbiota is crucial in determining respiratory health and preventing colonization of respiratory pathogens. Infections of URT may be multifactorial and involve many pathogens, and may develop on courses influenced by a combination of factors. The primary bacterial species in the URT that may adversely affect avian health are *Ornithobacterium rhinotracheale*, *Mycoplasma synoviae*, *Mycoplasma gallisepticum*, *Gallibacterium anatis*, and *Escherichia coli* [11,27,28,55]. Detection of the first three of these was via the mgc2, 16–23S rRNA regions and 16S RNA gene identification by real-time PCR, respectively. The fourth, *G. anatis*, was identified by matrix-assisted laser desorption/ionization–time-of-flight mass spectrometry, by which method *Enterococcus faecium* and *E. faecalis* were also identified. Some of these microbial species are commonly found in both healthy and infected poultry respiratory tracts. The presence of these pathogens can affect the normal development of birds by reducing the productivity of the flock, although they did not cause other clinical signs in research subject birds [18,26,48,56]. However, under specific conditions (e.g., stress and viral infections) these species may cause symptoms of disease [11,25]. As in other studies describing the respiratory microbial ecosystem, in the present research both species of poultry also had potential pathogens identified in the URT that are commonly found there, such as *Ornithobacterium* spp., *Mycoplasma* spp., *Gallibacterium* spp., and *Avibacterium* spp. [17,18,48,49,57]. The *Mycoplasma* ASV prevalence in turkeys was lower and those ASV were present in two flocks of turkeys and 17 flocks of chickens. In contrast, *Ornithobacterium* ASV prevalence was higher in turkeys, noted in 16 flocks, and lower in hens, ascribed to three flocks. One of the most common bacterial genus was *Staphylococcus*, which is in agreement with other results for the poultry microbiota [18]. This genus was dominant in chickens, while *Enterococcus* was dominant in turkeys. Although there are known pathogens within the *Staphylococcus* genus, their presence in significant numbers in most flocks may suggest that a significant proportion of species behave as commensals. Subclinical pathogen occurrence is common in commercial chicken and turkey flocks but their presence could equally affect the productivity of birds (Figure 3 and Supplementary Tables S2 and S4). 

Differences in diversity and abundance between birds may be related to environmental factors such as biosecurity levels, housing, bedding or systematic changes such as changes in feeding or management. The age of birds is also one of the factors affecting respiratory microbiota (Figure 5c,d). As the turkeys aged, the number of unique observed ASV was higher in each sample (Figure 5c). In chickens, the increase in the number of observed unique ASV was higher with the commencement of chickens’ laying; this activity is a stressor which could weaken the bird’s immune system and allow colonization of the respiratory system (Figure 5d) [18,49,58]. Significant differences in observed ASV between chickens and turkeys were observed (*p* < 0.05, Kruskal–Wallis test). Seasonal differences were another factor influencing the occurrence of feed-borne and environmental pathogens. In this study, poultry farms were randomly selected from different geographical regions of Poland and the samples were from birds of different ages and therefore at different stages of laying. Diet and intervention strategies were generally consistent throughout this study. No antibiotics were used in any of the flocks studied and no major disease outbreaks occurred during the sampling period. Further factors possibly lying under the differences between birds were sex, breed, and flock cycle.

## 5. Conclusions

Studies of the microbial composition of chickens and turkeys have provided us with data for further research to better understand the poultry respiratory microbiome. The results obtained allowed us to analyze the bacteriological composition of the respiratory tracts of chickens and turkeys of different ages and from different geographic locations in Poland. The presence of some potential respiratory pathogens in turkeys like *Ornithobacterium rhinotracheale* may be the cause of the lower body weight of birds similarly to the presence of *Mycoplasma* spp. in chickens. Exploring the URT of chickens and turkeys will help us better understand the role of the respiratory microbiota in the health, welfare, and productivity of birds.

## Figures and Tables

**Table 1:** Information on samples collected from chickens and turkeys yielding DNA of sufficient quality for further analysis.

Chickens				Turkeys			
ID of Sample	Age (Weeks)	Year of Sampling	Location	ID of Sample	Age (Weeks)	Year of Sampling	Location
Ch-1	45	2020	Warmińsko-Mazurskie	T-1	11	2020	Wielkopolskie
Ch-2	45	2020	Warmińsko-Mazurskie	T-2	11	2020	Wielkopolskie
Ch-3	45	2020	Warmińsko-Mazurskie	T-3	48	2020	Warmińsko-Mazurskie
Ch-4	29	2020	Warmińsko-Mazurskie	T-4	48	2020	Warmińsko-Mazurskie
Ch-5	29	2020	Warmińsko-Mazurskie	T-5	21	2019	Warmińsko-Mazurskie
Ch-6	29	2020	Warmińsko-Mazurskie	T-6	21	2019	Warmińsko-Mazurskie
Ch-7	22	2019	Warmińsko-Mazurskie	T-7	37	2019	Lubelskie
Ch-8	18	2019	Wielkopolskie	T-8	3	2019	Wielkopolskie
Ch-9	26	2020	Warmińsko-Mazurskie	T-9	6	2019	Wielkopolskie
Ch-10	26	2020	Warmińsko-Mazurskie	T-10	3	2019	Śląskie
Ch-11	26	2020	Warmińsko-Mazurskie	T-11	50	2020	Warmińsko-Mazurskie
Ch-12	27	2020	Warmińsko-Mazurskie	T-12	21	2019	Lubelskie
Ch-13	27	2020	Warmińsko-Mazurskie	T-13	36	2019	Kujawsko-Pomorskie
Ch-14	27	2020	Warmińsko-Mazurskie	T-14	2	2020	Podlaskie
Ch-15	42	2020	Warmińsko-Mazurskie	T-15	49	2020	Warmińsko-Mazurskie
Ch-16	42	2020	Warmińsko-Mazurskie	T-16	49	2020	Warmińsko-Mazurskie
Ch-17	42	2020	Warmińsko-Mazurskie	T-17	49	2020	Warmińsko-Mazurskie
Ch-18	34	2020	Warmińsko-Mazurskie	T-18	30	2019	Warmińsko-Mazurskie
Ch-19	34	2020	Warmińsko-Mazurskie	T-19	36	2019	Warmińsko-Mazurskie
Ch-20	34	2020	Warmińsko-Mazurskie	T-20	21.5	2019	Śląskie
Ch-21	21	2020	Warmińsko-Mazurskie	T-21	8	2019	Kujawsko-Pomorskie
Ch-22	21	2020	Warmińsko-Mazurskie	T-22	20	2019	Warmińsko-Mazurskie
Ch-23	21	2020	Warmińsko-Mazurskie	T-23	13	2019	Warmińsko-Mazurskie
Ch-24	27	2019	Podkarpackie	T-24	13	2019	Warmińsko-Mazurskie
Ch-25	22	2019	Wielkopolskie	T-25	13	2019	Warmińsko-Mazurskie
Ch-26	22	2019	Wielkopolskie	T-26	30	2020	Warmińsko-Mazurskie
Ch-27	25	2020	Lubelskie				
Ch-28	25	2020	Lubelskie				

## Data Availability

The data presented in this study are available on request from the corresponding author. The data are not publicly available due to legislation protecting privacy.

## Supplementary Materials

The following supporting information can be downloaded at: https://www.mdpi.com/article/10.3390/microorganisms10050987/s1, Supplementary Tables S1–S4.
